# Transferrin receptors

**DOI:** 10.1038/s12276-025-01436-x

**Published:** 2025-04-22

**Authors:** Qian Guo, Christopher Qian, Xinyu Wang, Zhong-Ming Qian

**Affiliations:** 1 Laboratory of Drug Delivery, School of Medicine, Shanghai University, Shanghai, China; 2 School of Biomedical Sciences and Gerald Choa Neuroscience Centre, Faculty of Medicine, The Chinese University of Hong Kong, Shatin, Hong Kong; 3 National Clinical Research Center for Aging and Medicine, Huashan Hospital, Fudan University, Shanghai, China

**Keywords:** Endocytosis, Homeostasis

## Abstract

The transferrin receptor (TfR) is one of the key proteins involved in cellular iron uptake. TfR-mediated endocytosis of transferrin-bound iron is the major pathway for iron acquisition by most cells in the body. Over the past three decades, the studies on TfR have made significant progress, and also, our knowledge on cell iron uptake has greatly been improved. Here we focus on recent advances in the studies on TfR and a brief discussion of the structures and functions of four different types of TfR, namely TfR1 (transferrin receptor 1), TfR2 (transferrin receptor 2), TfR3 (glyceraldehyde-3-phosphate dehydrogenase) and TfR4 (cubilin). These proteins work in different cells or organs and at different times, ensuring that cells and tissues get the iron they need. Their normal expression and function are fundamental to the body’s iron homeostasis.

## Exploring the role of transferrin receptors in cellular iron homeostasis

Iron metabolism disorders affect many people worldwide, making it crucial to understand how the body manages iron. Here the authors review research on transferrin receptors (TfRs), which are proteins that help cells take in iron. The study focuses on four types of TfR: TfR1, TfR2, TfR3 and TfR4. TfR1 is the main receptor for iron uptake in most cells, especially red blood cells. It binds to transferrin and helps transport iron into cells. TfR2 is similar to TfR1 but plays a role in regulating hepcidin, a hormone that controls iron levels in the body. TfR3 and TfR4 are less understood but involved in rapid iron uptake during stress and in kidney function. The research highlights the importance of these receptors in maintaining iron balance. Future research could lead to new treatments for disorders caused by abnormal iron metabolism.

This summary was initially drafted using artificial intelligence, then revised and fact-checked by the author.

## Introduction

Iron metabolism disorders are a worldwide problem affecting billions of people^1^. Therefore, studying the homeostasis mechanism involved in body iron metabolism is essential to elucidate the pathophysiological mechanisms of iron metabolism disorders and to develop drug or pharmacological interventions to disrupt the pathological event chain of diseases caused by abnormal iron metabolism.

Although all genes involved in iron transport and metabolism have yet to be identified^2^, great progresses obtained during the past three decades about the discovery of divalent metal transporter (DMT1/DCT1/Nramp 2)^3,4^, ferroportin 1 (Fpn1/ IREG1/Slc40a1/MTP1)^5–7^, hepcidin (HAMP/LEAP-1)^8–11^, hephaestin^12^ and the potential role of cystathionine beta synthase^13,14^ and apolipoprotein E^15–18^ in the studies of iron homeostasis have made important contributions to understanding iron transport and metabolism in the human body.

Transferrin receptor (TfR) is one of the key proteins involved in cellular iron uptake. TfR-mediated transferrin-binding iron (Tf-Fe, also known as holo-Tf) endocytosis is the primary pathway by which iron is acquired by most cells in the body and also transported across the blood–brain barrier (BBB)^19–23^. The accumulated results from the studies on TfR have contributed significantly to our understanding of cellular iron uptake. This review focuses on the recent advances in the study of TfRs and briefly discusses the structure, expression and function of TfR1, TfR2, TfR3 (glyceraldehyde-3-phosphate dehydrogenase, GAPDH) and TfR4 (cubilin).

## TfR1

TfR1 (also known as CD71 or TfRC) is a type 2 transmembrane glycoprotein expressed as an homodimer with a molecular weight of 97 kDa (refs. ^24–26^). TfR1 is synthesized in the endoplasmic reticulum and modified by phosphates and fatty acyl groups after translation^27^.

The molecule consists of two identical monomers; each connected by two disulfide bonds at Cys89 and Cys98 (ref. ^28^) and can be divided into three segments: a short N_2_H terminal cytoplasmic region (residues 1–67), a single transmembrane pass (residues 68–88) and a large extracellular portion (ectodomain, residues 89–760), which is soluble and bears a trypsin sensitive site^25,26^. The short intracellular region contains a YXXφ internalization motif (Y20TRF23), the large extracellular section contains a binding site for transferrin^25,26^, and three N-chain glycosylation sites at Asn251, Asn317 and Asn727 and one O-chain glycosylation site at Thr104 (ref. ^27^), which are thought to be critical for TfR1 function. Mutations at the N-linked glycosylation sites impair transferrin-binding activity. Similarly, elimination of the O-linked glycosylation at Thr104 enhances the cleavage of TfR1 and promotes the release of its outer membrane domain^29^.

Crystallographic studies of the ectodomain of human TfR1 (residues 122–760) revealed that homodimer of TfR1 is organized as a butterfly-like shape. Each TfR1 monomer consists of three distinct globular domains^30^, identified as the protease-like, apical and helical domains, and form a lateral cleft, which is likely to be in contact with the docked transferrin molecules. The ectodomain of TfR1 is separated from the membrane by a stalk, which probably includes residues involved in disulfide bond formation and the O-linked glycosylation. The amino acid sequence of the globular ectodomain of TfR1 is 28% identical to that of membrane glutamate carboxypeptidase II, which hydrolyzes the most prevalent mammalian neuropeptide, *N*-acetyl-l-aspartyl-l-glutamate^30^.

Tf-Fe (or holo-Tf) uptake is the primary pathway by which most cells, especially developing red blood cells, acquire iron. Because the amount of iron entering the cell depends on the amount of TfR1 molecules on the cell surface, Tf-Fe uptake is also a rate-limiting step in iron entry into the cell and is critical in preventing iron overload. The binding of TfR1 to Tf is pH dependent. At pH 7.4, TfR1 binds Tf-Fe with high affinity of *K*_d_ around 10^−9^ mol l^−1^ (refs. ^31,32^), about 500 times higher than that for the iron-free transferrin (apo-Tf), so TfR1 binds to iron-saturated holo-Tf but not to iron-free apo-Tf on the cell surface. By contrast, TfR1 binds to apo-Tf but not holo-Tf when the pH is low in the endosome^33^, because the affinity of the former is much higher than that of the latter. TfR1 also binds to the hereditary hemochromatosis protein HFE^34^, and the binding of TfR1 with HFE reduces the receptor affinity for Tf^35^.

**Fig. 1:**
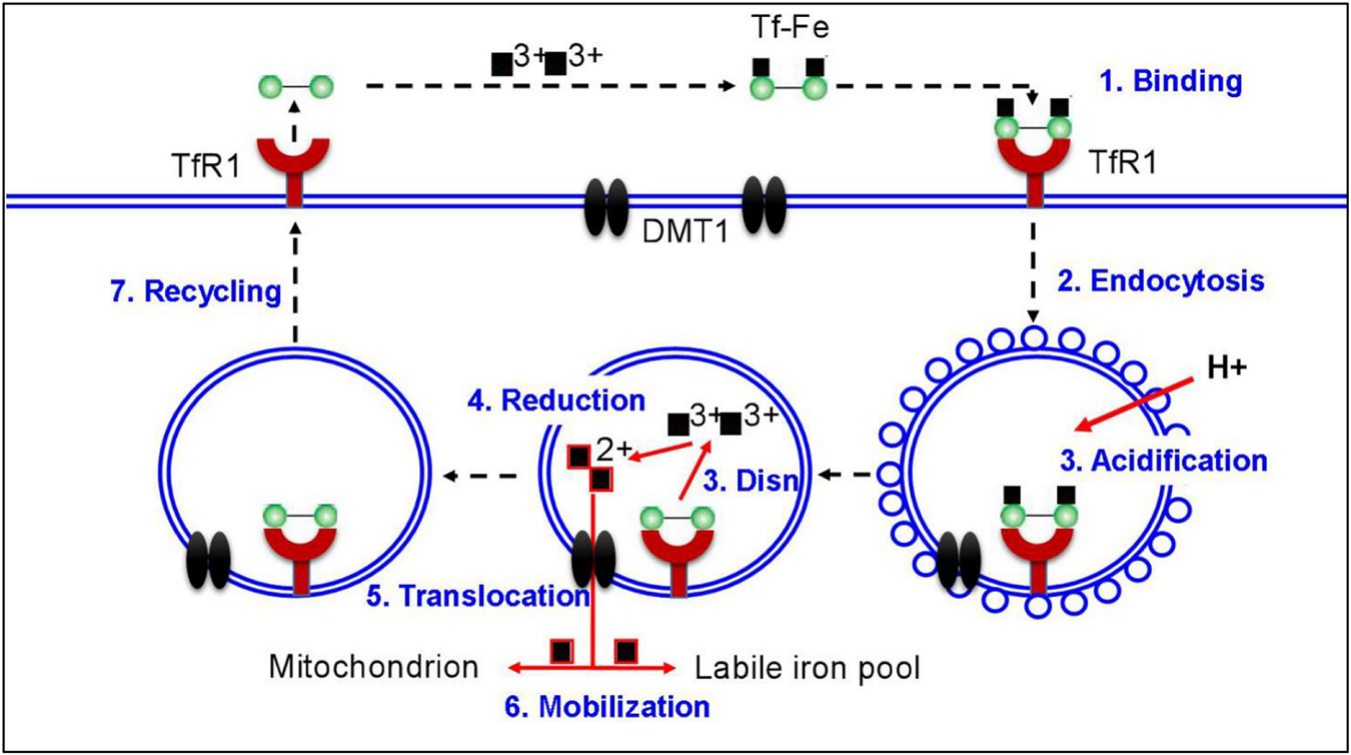
TfR1-mediated cellular iron uptake. This process can be divided into seven steps: (1) ‘binding’: two holo-Tf molecules bind to the dimeric TfR1; (2) ‘endocytosis’: clathrin-dependent endocytosis of the holo-Tf–TfR1 complex; (3) ‘acidification and dissociation (disn)’: the pH in the endosome is reduced to about 6.5 by the action of an H^+^-ATPase and Fe^3+^ dissociated from Tf; (4) ‘reduction’: Fe^3+^ is reduced to Fe^2+^ in the endosome, probably by Dcytb and STEAP2; (5) ‘translocation’: Fe^2+^ transports across the endosomal membrane by a process mediated by DMT1 or ZIP14; (6) ‘mobilization of iron for metabolism’: iron mostly transports into mitochondria or the cell’s labile iron pool; and (7) ‘recycling’: non-iron-bound Tf (apo-Tf) that remains associated with TfR1 in the recycling endosome is then transported to the cell surface, where apo-Tf is released into the blood stream at a pH of 7.4 (refs. ^23,36,37^).

The process of Tf-Fe uptake can be divided into seven steps^23,36,37^. The first step is ‘binding’: Fe2-Tf to the extracellular portion of TfR1 on the cell membrane^36,37^; the second is ‘endocytosis’: clathrin-dependent endocytosis of the holo-Tf–TfR1 complex^30^ (also, TfR1 continues to endocytose with or without Tf binding); the third is ‘acidification and dissociation’: the pH in the endosome is reduced to about 6.5 by the action of an H^+^-ATPase^38^ and Fe^3+^ dissociated from Tf; the fouth is ‘reduction’: Fe^3+^ is reduced to Fe^2+^ in the endosome, probably by the ferrireductases duodenal cytochrome b (Dcytb) and six-transmembrane epithelial antigen of the prostate 2 (STEAP2)^39,40^; the fifth is ‘translocation’: Fe^2+^ transports across the endosomal membrane by a process mediated by divalent metal transporter 1 (refs. ^3,4,22,41^) or ZIP14 (Zrt-like and Irt-like protein 14 or SLC39A14)^42^; the sixth is ‘mobilization of iron for metabolism’: iron, may be chaperoned by poly(rC)-binding protein 1 (ref. ^43^), mostly transports into mitochondria via the inner membrane protein mitoferrin 1–solute carrier family 25, member 37 (ref. ^44^) and/or the siderophore 2,5-dihydroxybenzoic acid^45^ for the synthesis of heme and iron–sulfur clusters^46^, with excess amounts being stored in the cytosol within the iron-storage protein ferritin^37^ or the cell’s labile iron pool^37^; and the seventh is ‘recycling’: apo-Tf that remains associated with TfR1 in the recycling endosome is then transported to the cell surface (and some of it will be degraded during this step), where apo-Tf is released into the blood stream at a pH of 7.4 (ref. ^40^) (Fig. 1). In erythroid cells, sorting nexin 3 was found to be required for the sorting of Tf–TfR1 complexes into recycling endosomes and exocyst complex component 6 for trafficking of the Tf–TfR1 complex from recycling endosomes to the cell surface^47–49^. It is highly probable that these two molecules may also play the same function in other types of cell. In addition, most cells have the ability to acquisition Fe^2+^ or nontransferrin-bound iron via a divalent metal transporter 1 (DMT1)-mediated pathway and to release iron via a Fpn1/Heph- and/or Fpn1/CP-mediated process.

TfR1 can also mediate cellular uptake of H-ferritin (not L-ferritin), an iron storage protein through endocytosis^50^. An interaction between TfR1 and H-ferritin requires more than a certain threshold level of TfR1 expression on the cell surface^51^. Thus, more than one TfR1 complex may be required for uptake of H-ferritin^51^. The erythroblasts that express very high levels of TfR1 can specifically incorporate H-ferritin, whereas peripheral lymphocytes and granulocytes cannot^51^. In contrast to Tf-Fe, H-ferritin dissociates from TfR1 in endosomes and are routed to lysosomes for degradation^50^. The physiological role of TfR1–H-ferritin uptake remains to be elucidated.

In addition, it has been reported that TfR1 is also one of the partner proteins involved in the antimetastatic effects of anti-CD81 antibody (5A6)^52^ and that TfR1 recycling is a revolving door mechanism exploited by influenza A virus to enter host cells^53^. TfR1 is also an entry receptor for many related human pathogens, such as New World arenavirus^54^, *Plasmodium vivax*^55^ or rabies virus^56^, and the role of TfR1 in influenza A virus entry is distinct from the role in the entry of New World arenavirus or *Plasmodium vivax*, in which the well-defined protein–protein interfaces between the pathogen surface proteins and TfR1 were identified by structural approaches. By disrupting the expression of the TfR1 gene in oligodendrocyte progenitor cells on mice using the *Cre/lox* system, it was found that TfR1 is necessary for proper iron homeostasis and development in oligodendrocyte^57^. Hepatocyte TfR1 has also been found to play a role in iron homeostasis by interacting with the hereditary hemochromatosis protein HFE to regulate hepcidin expression^58^. Tf-Fe uptake has been shown to be particularly required for osteoclast function and indispensable for bone remodeling in a gender-dependent manner^59^.

The TfR1 has also been used to increase transport of antibody-based therapeutics across the BBB. A number of such ‘engineered antibodies’ modified into bi-specific formats have been developed that have the ability to enter the brain and approach pathological proteins, such as amyloid-beta, through TfR1-mediated endocytosis^60^. Recently, there has been a growing effort to develop promising gene vectors for the treatment of brain-related diseases. One such development is the self-assembled H-ferritin nanoparticles that can encapsulate nucleic acid drugs and specifically bind to BBB endothelial cells via interactions with the TfR1, thereby increasing uptake through the BBB^61^. A functional selection method has been established to identify high affinity single domain antibodies to the TfR1 with efficient biotherapeutic delivery across the BBB^62^. In addition, the increased expression of TfR1 observed in malignant cells make this receptor an attractive target for antibody-mediated cancer therapy and a mouse/human chimeric IgG3 specific for human TfR1 (ch128.1, an antibody-avidin fusion protein) has developed, which shows antitumor activity against certain malignant B cells in vitro through TfR1 degradation and iron deprivation^63^.

## TfR2

TfR2 is an 89 kDa type II transmembrane glycoprotein that contains 801 amino acids with 45% homology and 66% similarity with TfR1 in the extracellular domain^64^. It is encoded by a 2,471-bp-long gene located on the long arm of human chromosome 7 (7q22.1) that consists of 18 exons^64^. This gene expresses two transcripts: alpha (TfR2α, approximately 2.9 kilobase pairs) and beta (TfR2β, approximately 2.5 kilobase pairs).

TfR2α originates from the transcription of all exons. Similar to TfR1, TfR2ɑ has a short cytoplasmic tail (amino acids 1–80) containing a consensus sequence YQRV for endocytosis, a transmembrane domain (amino acids 81–104) with four cysteines (amino acids 89–98 and 108–111) and a large extracellular domain (amino acids 105–801) comprising a protease-associated domain and two RGD elements that bind Tf-Fe. The four cysteines in transmembrane domain involve in disulfide bonds that may cause TfR2 homo-dimerization^65^. Also, there is an N-terminal mitochondrial targeting sequence in TfR2ɑ intracellular domain^66^. Because TfR2ɑ does not possess an iron-responsive element^64^, therefore, the expression of TfR2ɑ should be not regulated by an iron regulatory protein-mediated process in response to cellular iron^64,67,68^ and may be controlled by other mechanisms, probably related to the cell cycle or cellular proliferation status^33,69^. The studies have also showed that the erythroid transcription factor GATA-1 could control TfR2ɑ expression at the transcriptional level^70^, while the hepatic tetraspanin CD81 is able to induce TfR2ɑ degradation by interacting with it^71^.

TfR2ɑ is predominantly expressed in hepatocytes and erythroid precursors, while TfR2β widely distributed and expressed at low levels and mostly expressed in spleen, heart and brain^64^. The β transcript lacks exons 1–3 and has an additional 142 nucleotide 5′-sequence in exon 4 but does not contain the start codon. Translation probably begins with the ATG at nucleotide 542, which is within the frame of the open reading frame of the transcript and contains a G at positions −3 and +4. The exons 1–3 encode the entire transmembrane and cytoplasmic domains, as well as a part of the extracellular domain, including the two cysteines at 108 and 111; therefore, the predicted protein product of the β transcript lacked both the transmembrane domain and the signal peptide, resulting in a possible intracellular protein^64^. Currently, very little is known about the transcriptional/translational regulatory pathways for TfR2β^65^.

TfR2 has a similar function to TfR1 with respect to Tf binding and Tf-mediated iron uptake. As TfR1, TfR2 interacts with Tf also in a pH-dependent manner. Apo-Tf binds to TfR2 only at acidic pH, while holo-Tf binds at neutral or higher pH^72^. In addition to Tf-Fe uptake by cells, TfR2 can also deliver Tf-Fe to mitochondria and to the respiratory complex I, playing a role as a mitochondrial iron transport system^66^. However, the affinity of TfR2 for Tf-Fe is 25- to 27-fold lower than that of TfR1 for Tf-Fe^32,64^ (Table 1). Also, mutations in TfR2 or lacking TfR2 in both human and mice are found to induce iron overload rather than iron deficiency in the liver^73^. These findings indicate that iron uptake by TfR2 into the liver may not be the primary function for this receptor^74^.

**Table 1:** The binding affinity of Tf to TfR1–4.

Tf binding to TfRs	Equilibrium dissociation constant (*K*_d_)	Ref.
Tf–TfR1	1 nM	Raje et al. (ref. ^87^)
Tf–TfR2	27 nM	West et al. (ref. ^30^)
Tf–TfR3 (GAPDH)	120 nM	Raje et al. (ref. ^87^)
Tf–TfR4 (cubilin)	20 nM	Kozyraki et al. (ref. ^102^)

*K*_d_ was determined using surface plasmon resonance.

**Fig. 2:**
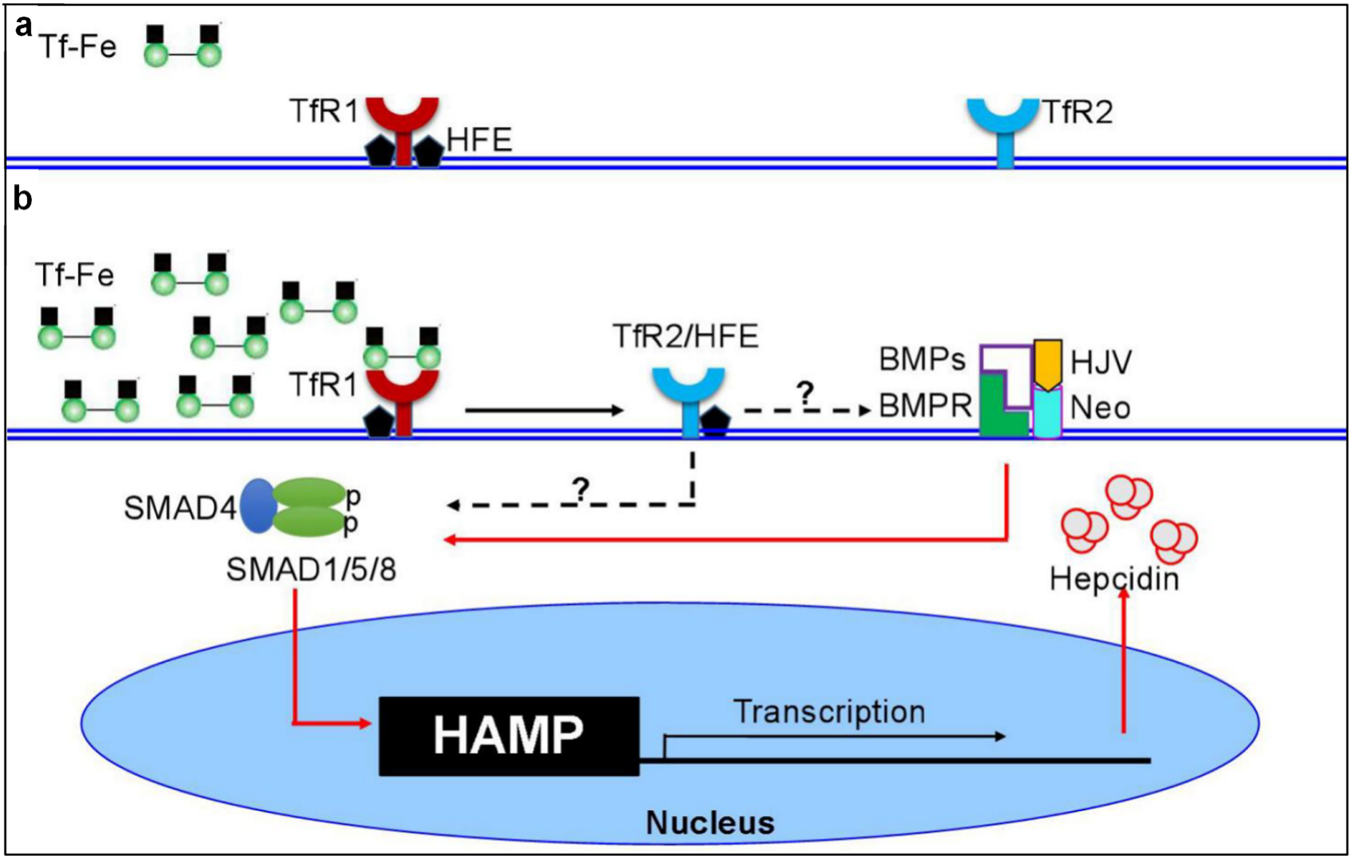
The most important function of TfR2 is to control the expression of hepcidin. **a** HFE (a hemochromatosis protein) sequestered by TfR1 at a low saturation of Tf-Fe in the serum. **b** At a high saturation of Tf-Fe in the serum, HFE is dislodged from its overlapping binding site on TfR1 by Tf-Fe and then is free to interact with TfR2 on the surface of hepatocytes. The formed complex of TfR2 and HFE interacts with HJV, BMPs (secreted by hepatic sinusoidal epithelial cells) and neogenin (Neo) signals, thereby initiating intracellular signaling toward the expression of hepcidin through phosphorylation of SMAD proteins. The phosphorylated SMAD (pSMAD) protein complex is translocated to the nucleus and binds to the BMP-responsive elements in the gene promoter of hepcidin (HAMP)^76,78^.

Of all the functions of TfR2, controlling the expression of hepcidin is the most important. It has been demonstrated that TfR2 is one of the hepcidin regulators, playing a key role in regulating iron homeostasis in the body^74^. At low saturation of Tf-Fe in the serum, HFE (a hemochromatosis protein) was sequestered by TfR1 (Fig. 2a). At high saturation of Tf-Fe in the serum, HFE is dislodged from its overlapping binding site on TfR1 by Tf-Fe, which competes with HFE to bind to TfR1, and then free to interact with TfR2 on the surface of hepatocytes^75,76^ (Fig. 2b). The complex of TfR2 and HFE serves as a component of the iron sensing machinery in hepatocytes to initiates regulation of hepcidin expression^75^. It has also been identified that there are two bone morphogenetic protein (BMP)-responsive elements in the promoter region of hepcidin^75^.

When the saturation of Tf-Fe in the serum increases, the formed complex of TfR2 and HFE interacts with hemojuvelin (HJV), a glycosylphosphatidylinositol anchored membrane protein and a coreceptor for BMPs, which belong to the transforming growth factor-β superfamily^77^. Also, hepatic sinusoidal epithelial cells secrete BMP6 and BMP2, both as homo- and heterodimers, which interact with the BMP type I and II receptors together with TfR2–HFE–HJV and neogenin (Neo) signals, thereby initiating intracellular signaling toward the expression of hepcidin through phosphorylation of SMAD proteins^75,78^. The phosphorylated SMAD protein complex is translocated to the nucleus and binds to the BMP-responsive elements in the gene promoter of hepcidin (HAMP)^75,78^ (Fig. 2b). In this process, TfR2 and HFE have a prevalent role of iron sensors and reinforce the BMP signaling, while HJV has the role of BMP coreceptor. In addition to the BMP–SMAD signaling pathway, the p38MAPK–ERK1/2 pathway has also been suggested to be involved in the TfR2–HFE complex regulation of hepcidin expression, because the levels of both pERK1/2 and phosphorylated SMAD are low in TfR2- and HFE-double-knockout mice^79^. However, p38MAPK–ERK1/2 activation was not detected in acute and chronic iron load mouse models, making the role of the p38MAPK–ERK signaling pathway controversial^80^.

In addition, TfR2 is a mediator of iron-erythropoietic cross-talk and its deletion in the liver hampers hepcidin production, increasing iron absorption, whereas its deletion in the hematopoietic compartment increases erythropoietic EPO sensitivity and erythropoietic production^81^. TfR2 could form a complex with the erythropoietin receptor (EpoR) and regulates EPO signaling and erythropoiesis in erythroid cells^29,82^. TfR2 has also been found to be expressed in osteoclasts and osteoblasts, acts as an inhibitor of bone formation by binding to BMP2 (as a receptor for BMP2) and inducing the expression of the Wnt inhibitor sclerostatin via the p38MAPK signaling pathway in bone^83^. TfR2 in macrophages has been found to have a protective role on the progression of arthritis by inhibiting M1 (a proinflammatory state)-like polarization^84^.

## TfR3 (GAPDH)

Williams and coworkers were the first to report that *Staphylococcus aureus* and *Staphylococcus epidermidis* express a receptor for human transferrin, a 42-kDa cell wall transferrin-binding protein that is involved in the acquisition of transferrin-bound iron^85^. They characterized this protein further and demonstrated that the staphylococcal TfR protein is a multifunctional cell wall GAPDH^86^. Raje et al. ^87^ showed for the first time that GAPDH is a novel TfR localized on the surface of human and mouse macrophages. They demonstrated that GAPDH expression is modulated by the availability of iron in the medium and that the GAPDH-Tf complex is subsequently internalized into early endosomes. Later studies have found that this ‘third receptor for Tf’^88,89^ is also expressed in CHO-TRVb cell lines lacking TfR1 and TfR2 (ref. ^90^) and on the surface of various cell types^88,89^.

Mammalian GAPDH is a ~150 kDa glycolytic enzyme composed of four identical 37 kDa subunits^88^ with approximately 2,000,000 molecules per cell and a molar concentration of approximately 0.4 µM (ref. ^91^). Similar to many other moonlighting or multifunctional proteins, GAPDH has no structural similarity to TfR1 or TfR2 nor does it contain membrane-targeting sequences^92^. GAPDH is located in not only on plasma membrane but also in the cytoplasm and the nucleus^93,94^. Owing to differential cellular localization, GAPDH has a vast diversity of functions^91,95^. GAPDH regulates microtubule bundling, actin polymerization, membrane fusion and vesicular trafficking^96^ in the cytosol, plays a key role in apoptosis and autophagy^96,97^ and maintenance of telomere length in the nucleus, and is also involved in the heme maturation of myoglobin and hemoglobin^98^.

**Fig. 3:**
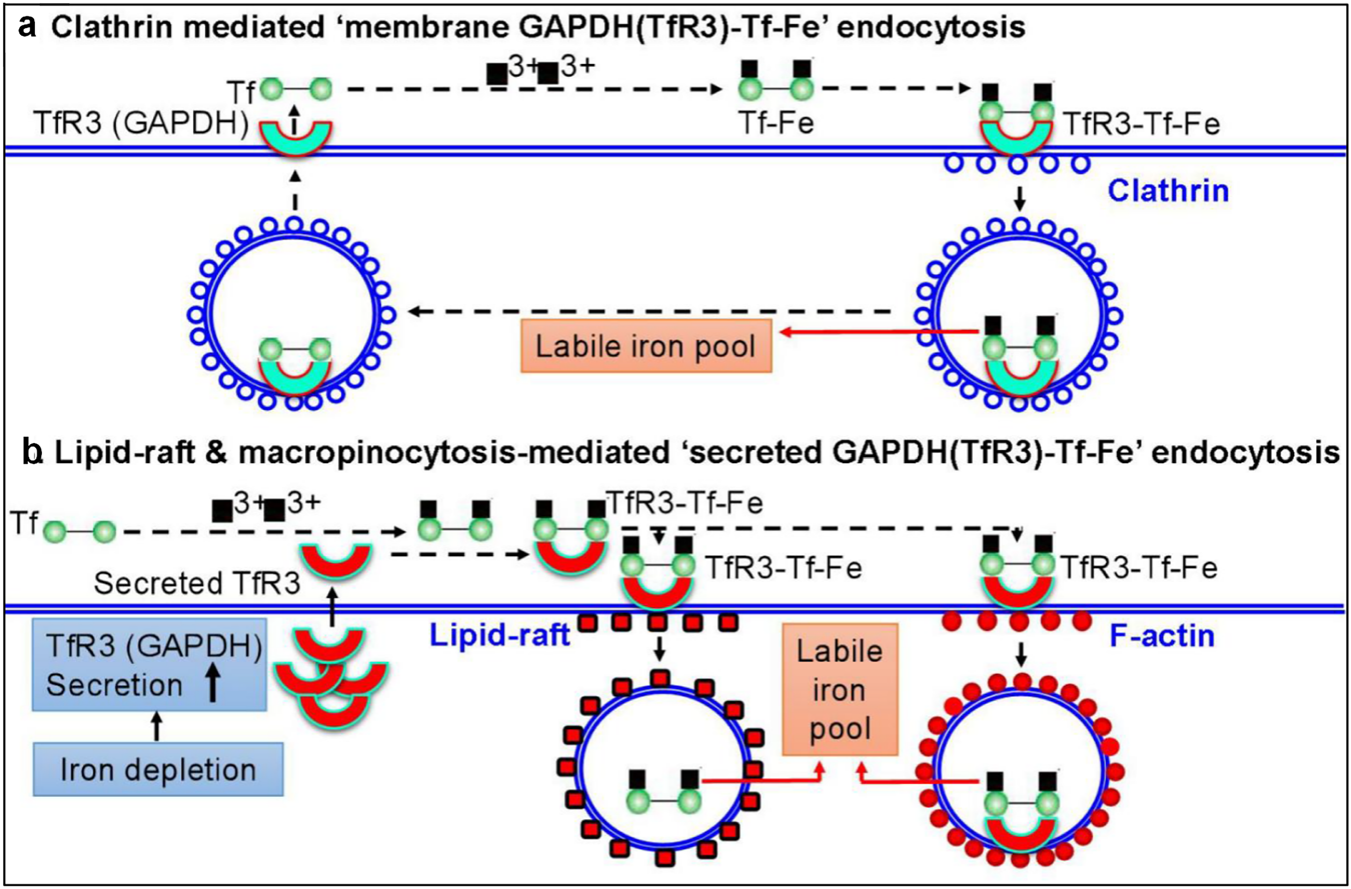
GAPDH on plasma membrane functions as the third TfR to mediate Tf-Fe uptake. **a**, **b** The complex of GAPDH with Tf-Fe is internalized by not only clathrin-mediated endocytosis (**a**) but also lipid-raft endocytosis (**b**). The binding of GAPDH (TfR3) with Tf-Fe is a low-affinity, saturable and pronase-sensitive process. The localization of GAPDH at the cell surface is regulated by cellular iron levels. Iron depletion causes the post-translational modification and recruitment of GAPDH to the membrane by unelucidated events. GAPDH could also be secreted by mammalian cells and the sGAPDH constitutes a normal component of serum and can also enhance the uptake of Tf-Fe into various mammalian cell types^90^.

Raje and colleagues^90^ suggested that transferrin-bound iron can be acquired by three TfRs: TfR1, TfR2 or GAPDH, depending on the cell type, and GAPDH on plasma membrane functions as the ‘third TfR’^90^ or ‘TfR3’^88^ to mediate Tf-Fe uptake. The binding of GAPDH with Tf-Fe is a low-affinity, saturable and pronase-sensitive process^88^. The localization of GAPDH at the cell surface is regulated by cellular iron levels. Iron depletion causes the post-translational modification and recruitment of GAPDH to the membrane by unelucidated events. Unlike TfR1–Tf-Fe and TfR2–Tf-Fe^99^, the complex of GAPDH with Tf-Fe is internalized by not only clathrin-mediated endocytosis but also lipid-raft endocytosis^90^ (Fig. 3). GAPDH could also be secreted by mammalian cells^87^, and the secreted GAPDH (sGAPDH) constitutes a normal component of serum^100^. In addition to GAPDH on the cell surface, sGAPDH can also enhance the uptake of Tf-Fe into various mammalian cell types, functioning as a soluble TfR, and urokinase plasminogen activator receptor (uPAR or CD87), a raftlocalized molecule, may be involved in sGAPDH-mediated Tf-Fe uptake^101^.

The physiological significance of TfR3 (GAPDH and sGAPDH)-mediated Tf-Fe has not been completely elucidated. Recent studies demonstrated that GAPDH-mediated Tf-Fe uptake is a rapid-response mechanism by which cells acquire iron during the early stages of hypoxia before specialized receptors such as TfR1 and TfR2 can be synthesized and deployed to the cell membrane^99^. Also, iron depletion was found to cause cells not only to recruit more GAPDH to their surface but also to enhance its secretion^94,101^. Therefore, it is highly probable that TfR3 (GAPDH and sGAPDH) may be a cellular rapid-response molecule for maintenance of iron homeostasis under stress conditions such as hypoxia^94^.

## TfR4 (cubilin)

Using a cubilin-affinity approach, Kozyraki et al.^102^ discovered Tf as a novel ligand to cubilin, and subsequently, they investigated the receptor-mediated uptake of Tf in the renal proximal tubules and in cultured yolk cells and demonstrate that cubilin is a physiological and quantitatively important ‘third Tf receptor’ and that cubilin-mediated endocytosis is a major pathway for the apical uptake of Tf in the renal proximal tubule cells. In addition to Tf and vitamin B_12_, this protein has also been reported to function as a receptor for apolipoprotein A1 and a low-affinity albumin receptor in the kidney proximal tubules and/or the yolk sac^103–106^. Since GAPDH has been called the ‘third TfR’^90^, ‘TfR3’^88^ or ‘third receptor for Tf’^88,89^ to mediate Tf-Fe uptake^90^, and the first paper on GAPDH as a receptor for human transferrin was published in the year 1994 (ref. ^85^), slightly before the paper published by Kozyraki et al. in the year 2001 (ref. ^102^), we suggest that cubilin be called TfR4.

Cubilin, a multiligand receptor structurally distinct from the presently known TfRs, is a large membrane glycosylated protein (460 kDa) with a unique set of extracellular protein modules comprising eight tandem epidermal growth factor domains followed by 27 tandem CUB domains (complement components C1r/C1s, Uegf (epidermal growth factor-related sea urchin protein) and bone morphogenic protein 1)^103,104,107^. The numerous CUB domains are ligand binding, and each consists of ~120 residues^108^. Cubilin has higher expression in the apical membrane of kidney proximal tubule and rodent yolk sac epithelial cells^109,110^ and in rat and human podocytes^111^ besides expression in the intestine.

**Fig. 4:**
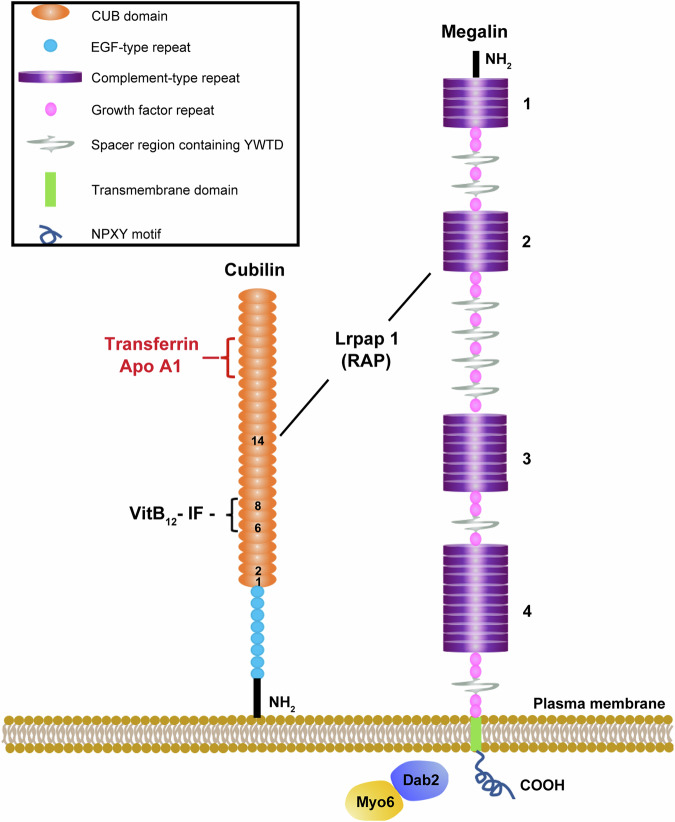
A cartoon diagram of the structure of the cubilin–AMN–megalin multiligand receptor endocytic complex. Cubilin is a peripheral membrane receptor composed of a short amino terminal, eight EGF type domains and 27 CUB domains (orange). The cubilin ligands include transferrin (Tf), apolipoprotein A1 (ApoA1), albumin, hemoglobin and intrinsic factor (IF)-vitamin B_12_ (VitB_12_). Megalin is a cell-surface receptor/transporter consisting of a large extracellular region, a single transmembrane domain and a C-terminal cytoplasmic tail. The extracellular domain of megalin contains four clusters of lipoprotein receptor ligand-binding repeats (purple), growth factor repeats, EGF repeats and YWTD spacer regions. The cytoplasmic tail of megalin binds Dab2, a cytosolic adapter protein important for megalin-mediated endocytosis, and Dab2 binds and recruits Myo6 to clathrin-coated vesicles. The receptor-associated protein (Lrpap1; RAP) binds both megalin and cubilin^113,114^.

Cubilin lacks a transmembrane and a cytoplasmic domain and the membrane trafficking of cubilin must be assisted by megalin, which colocalizes with and binds to cubilin^104,112^. Cubilin interacts with megalin to form a cubilin–megalin complex or cubilin–megalin tandem receptor^110,113^ (Fig. 4). In fact, the internalization of the cubilin–Tf complex is accomplished by the cubilin–megalin tandem receptor, rather than cubilin alone. The mice with deficient synthesis of megalin fail to internalize Tf in their proximal tubules, indicating an essential role of megalin in the endocytosis of the cubilin–Tf complex^104^.

Megalin was first identified by Kerjaschki and Farquhar^114,115^ and is a 600 kDa (4,655 amino acids) single transmembrane domain receptor protein that belongs to the low-density lipoprotein receptor family^107,116^. Megalin is responsible for the normal proximal tubule reabsorption of filtered plasma proteins, thus preventing the loss of these essential molecules into the urine^110^. The almost complete clearance of proteins from the ultrafiltrate by megalin-driven endocytosis is accomplished in cooperation with the cubilin.

**Fig. 5:**
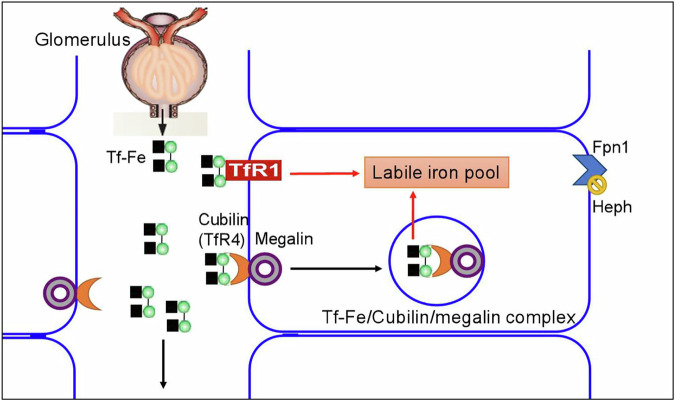
Cubilin-mediated endocytosis is a major pathway for the apical uptake of Tf-Fe in the renal proximal tubule cells. The filtered TF-Fe is reabsorbed in the renal proximal tubules by binding to cubilin (TfR4) and then internalized. Because cubilin lacks a transmembrane and a cytoplasmic domain, the membrane trafficking of cubilin must be assisted by megalin, which colocalizes with and binds to cubilin. The internalization of the cubilin–Tf-Fe complex is accomplished by the cubilin–megalin tandem receptor, rather than cubilin alone. The cubilin–megalin-mediated uptake of Tf-Fe has been suggested to be a biological process for rescuing iron and for supplying the iron-dependent enzymes in the renal proximal tubules^102^.

Under physiologic conditions, nontransferrin-bound iron and Tf-Fe passes the glomerular filter and is reabsorbed through kidney epithelial cells^117^. Reabsorption of filtered Tf-Fe in the renal proximal tubules has been mainly attributed to the cubilin–megalin tandem receptor-mediated endocytosis^102^ (Fig. 5) and the cubilin–megalin-mediated uptake of Tf has been suggested to be a biological process for rescuing iron and for supplying the iron-dependent enzymes in the renal proximal tubules^102^. A pivotal role of the cubilin–megalin complex for Tf uptake in the kidney is demonstrated by the findings that cubilin- or megalin-deficiency could induce a complete lack of Tf reabsorption and a high excretion of Tf in the urine^102^. In kidneys, TfR1 expression was decreased while the cubilin–megalin tandem receptor was highly upregulated in mice treated with iron dextran injections, suggesting that the cubilin–megalin tandem receptor may effectively mediate reabsorption of Tf-Fe that cycles through the kidney during parenterally induced iron overload despite the reduction of TfR1 (ref. ^117^). In addition to iron^118^, expression and function of the cubilin–megalin complex has been reported to be also affected by age^119^, type 1 diabetes^120^, renal endosome-associated chloride channel (ClC-5)^121^ and heme oxygenase 1 (HO1)^122^.

The normal function of cubilin is also dependent on amnionless (AMN), a single transmembrane protein of 38–50 kDa, which is essential for the trafficking of cubilin to the apical plasma membrane^123,124^. AMN was originally identified by random mutagenesis as a protein essential for amnion and primitive streak formation in mice. AMN is highly expressed in cubilin-expressing tissues, including the kidney, intestine and mouse visceral yolk sac^123,124^. AMN and cubilin have a consistent tissue distribution and genetic abnormalities in AMN and cubilin have similar phenotypic consequences^123–125^. These suggest a functional link between the two gene products. Fyfe et al. ^107^ investigated the possibility that the two proteins interact and demonstrtaed that AMN and cubilin form a tightly bound complex early in the biosynthetic pathway that is essential for apical membrane localization and endocytic functions previously ascribed to cubilin alone. Currently, it is unclear whether AMN is involved in the internalization of the cubilin–Tf complex. However, the possibility exists and is worth investigating.

In addition to Tf and Tf-Fe, the megalin–cubilin complex also plays a role in taking up cadmium (Cd^2+^)–metallothionein complexes apart from transport by the ZIP transporters and DMT1 (ref. ^126^). The Cd^2+^–metallothionein complexes are the major delivery form of Cd^2+^, a nonessential divalent metal ion that causes toxicity in multiple organs including kidney^126^. The megalin–cubilin complex also contributes to the reabsorption or the uptake of radiolabeled somatostatin analogs via receptor-mediated endocytosis by renal proximal tubular cells^127^. The function of cubilin has also been reported to be dependent on a single transmembrane protein AMN^107^.

## Concluding remarks

Studies on TfR1, TfR2, TfR3 (GAPDH) and TfR4 (cubilin–megalin tandem receptor) that are reviewed in the preceding paragraphs have made important contributions for the understanding of the mechanisms by which iron uptake by different types of mammalian cell and have greatly improved our understanding of iron homeostasis in the body. The cumulative research evidence proves that all of these receptor proteins are moonlighting or multifunctional proteins (that have more than one function)^103^ and that mediating cell uptake of Tf-Fe is the common function of them. It has been well confirmed that TfR1 is a primary iron (Tf-Fe or holo-Tf) acquirer in most cells, especially developing red blood cells, while TfR4 is mainly responsible for iron uptake in the apical membrane of kidney proximal tubule where it is higher expressed. TfR2 has similar functions to TfR1 in Tf binding and Tf-mediated iron uptake in liver cells, erythroid precursors as well as in spleen, heart and brain, although its most important function is to control hepcidin expression, while TfR3-mediated Tf-fe uptake is a rapid-response mechanism by which cells acquire iron in the early stages of stress conditions before TfR1 and TfR2 are synthesized and deployed to the cell membrane. These proteins have different binding affinities to Tf (Table 1), work in different cells or organs and at different times, ensuring that cells and tissues get the iron they need. Their normal expression and function are fundamental to the body’s iron homeostasis.

However, it should be pointed out that the physiological importance of these TfRs, especially TfR3 and TfR4, in providing iron utilization to cells and tissues, as well as their role in iron homeostasis in the body, still requires further research to refine. Also, the regulatory mechanisms controlling the expression of these receptors have not been fully elucidated. In addition, further work is clearly needed to detail and confirm the functional mechanisms involved in the TfR3- and TfR4-mediated iron uptake pathways. Moreover, little is currently known about whether there are any functional links between these different TfRs-mediated iron uptake pathways. Furthermore, the pathophysiological implications of TfRs detailed herein are important, especially for diseases associated with the disrupted expression of these receptors, and further investigations on this important aspect is definitely needed. Finally, whether there are other unknown TfRs is also worth investigating. A full understanding of these suggested aspects is critical not only to elucidate the normal physiology of iron homeostasis and the pathophysiological mechanisms of TfRs-related iron metabolic disorders but also to develop pharmacological interventions capable of disrupting the chain of pathological events that occur in these disorders.

## Data Availability

Not applicable.

## References

[1] Altamura, S. et al. Regulation of iron homeostasis: lessons from mouse models. *Mol. Asp. Med* **75**, 100872 (2020). 10.1016/j.mam.2020.100872 32792212

[2] Roy, C. N. & Andrews, N. C. Recent advances in disorders of iron metabolism: mutations, mechanisms and modifiers. *Hum. Mol. Genet* **10**, 2181–2186 (2001). 11673399 10.1093/hmg/10.20.2181

[3] Gunshin, H. et al. Cloning and characterization of a mammalian proton-coupled metal-ion transporter. *Nature* **388**, 482–488 (1997). 9242408 10.1038/41343

[4] Fleming, M. D. et al. Microcytic anaemia mice have a mutation in *Nramp2*, a candidate iron transporter gene. *Nat. Genet* **16**, 383–386 (1997). 9241278 10.1038/ng0897-383

[5] Abboud, S. & Haile, D. J. A novel mammalian iron-regulated protein involved in intracellular iron metabolism. *J. Biol. Chem.* **275**, 9906–9912 (2000). 10.1074/jbc.M000713200 10747949

[6] Donovan, A. et al. Positional cloning of zebrafish ferroportin1 identifies a conserved vertebrate iron exporter. *Nature* **403**, 776–781 (2000). 10693807 10.1038/35001596

[7] McKie, A. T. et al. An iron-regulated ferric reductase associated with the absorption of dietary iron. *Science* **291**, 1755–1759 (2001). 11230685 10.1126/science.1057206

[8] Krause, A. et al. LEAP-1, a novel highly disulfide-bonded human peptide, exhibits antimicrobial activity. *FEBS Lett.* **480**, 147–150 (2000). 11034317 10.1016/s0014-5793(00)01920-7

[9] Park, C. H. et al. Hepcidin, a urinary antimicrobial peptide synthesized in the liver. *J. Biol. Chem.* **276**, 7806–7810 (2001). 11113131 10.1074/jbc.M008922200

[10] Pigeon, C. et al. A new mouse liver-specific gene, encoding a protein homologous to human antimicrobial peptide hepcidin, is overexpressed during iron overload. *J. Biol. Chem.* **276**, 7811–7819 (2001). 11113132 10.1074/jbc.M008923200

[11] Nicolas, G. et al. Lack of hepcidin gene expression and severe tissue iron overload in upstream stimulatory factor 2 (USF2) knockout mice. *Proc. Natl Acad. Sci. USA* **98**, 8780–8785 (2001). 11447267 10.1073/pnas.151179498 PMC37512

[12] Vulpe, C. D. et al. Hephaestin, a ceruloplasmin homologue implicated in intestinal iron transport, is defective in the *sla* mouse. *Nat. Genet* **21**, 195–199 (1999). 9988272 10.1038/5979

[13] Zhao, P. et al. Cystathionine β-synthase (CBS) deficiency suppresses erythropoiesis by disrupting expression of heme biosynthetic enzymes and transporter. *Cell Death Dis.* **10**, 708 (2019). 31551410 10.1038/s41419-019-1951-0 PMC6760157

[14] Zhou, Y. F. et al. Cystathionine β-synthase is required for body iron homeostasis. *Hepatology* **67**, 21–35 (2018). 28859237 10.1002/hep.29499

[15] Ma, J. et al. Apolipoprotein E deficiency induces a progressive increase in tissue iron contents with age in mice. *Redox Biol.* **40**, 101865 (2021). 33493903 10.1016/j.redox.2021.101865 PMC7823209

[16] Ma, J. et al. The role of iron in atherosclerosis in apolipoprotein E deficient mice. *Front. Cardiovasc. Med.* **9**, 857933 (2022). 35669479 10.3389/fcvm.2022.857933 PMC9163807

[17] Ma, J. et al. Apolipoprotein E is required for brain iron homeostasis in mice. *Redox Biol.* **64**, 102779 (2023). 37339558 10.1016/j.redox.2023.102779 PMC10363452

[18] Guo, Q., Qian, C. & Qian, Z. M. Iron metabolism and atherosclerosis. *Trends Endocrinol. Metab.* **34**, 404–413 (2023). 37210298 10.1016/j.tem.2023.04.003

[19] Qian, Z. M. & Wang, Q. Expression of iron transport proteins and excessive iron accumulation in the brain in neurodegenerative disorders. *Brain Res. Rev.* **27**, 257–267 (1998). 9729418 10.1016/s0165-0173(98)00012-5

[20] Qian, Z. M. & Shen, X. Brain iron transport and neurodegeneration. *Trend. Mol. Med* **7**, 103–108 (2001). 10.1016/s1471-4914(00)01910-9 11286780

[21] Ke, Y. & Qian, Z. M. Iron misregulation in the brain: a primary cause of neurodegenerative disorders. *Lancet Neurol.* **2**, 246–253 (2003). 12849213 10.1016/s1474-4422(03)00353-3

[22] Ke, Y. & Qian, Z. M. Brain iron metabolism: neurobiology and neurochemistry. *Prog. Neurobiol.* **83**, 149–173 (2007). 17870230 10.1016/j.pneurobio.2007.07.009

[23] Qian, Z. M. & Ke, Y. Brain iron transport. *Biol. Rev.* **94**, 1672–1684 (2019). 31190441 10.1111/brv.12521

[24] Kühn, L. C., McClelland, A. & Ruddle, F. H. Gene transfer, expression, and molecular cloning of the human transferrin receptor gene. *Cell* **39**, 5–103 (1984). 6327061 10.1016/0092-8674(84)90304-0

[25] McClelland, A., Kühn, L. C. & Ruddle, F. H. The human transferrin receptor gene: genomic organization, and the complete primary structure of the receptor deduced from a cDNA sequence. *Cell* **39**, 267–274 (1984). 6094009 10.1016/0092-8674(84)90004-7

[26] Schneider, C. et al. Primary structure of human transferrin receptor deduced from the mRNA sequence. *Nature* **311**, 675–678 (1984). 6090955 10.1038/311675b0

[27] Omary, M. B. & Trowbridge, I. S. Biosynthesis of the human transferrin receptor in cultured cells. *J. Biol. Chem.* **256**, 12888–12892 (1981). 6273413

[28] Jing, S. Q. & Trowbridge, I. S. Identification of the intermolecular disulfide bonds of the human transferrin receptor and its lipid-attachment site. *EMBO J.* **6**, 327–331 (1987). 3582362 10.1002/j.1460-2075.1987.tb04758.x PMC553399

[29] Moura, I. C. et al. Erythropoiesis and transferrin receptors. *Curr. Opin. Hematol.* **22**, 193–198 (2015). 25767952 10.1097/MOH.0000000000000133

[30] Lawrence, C. M. et al. Crystal structure of the ectodomain of human transferrin receptor. *Science* **286**, 779–782 (1999). 10531064 10.1126/science.286.5440.779

[31] Lebron, J. A. et al. Crystal structure of the hemochromatosis protein HFE and characterization of its interaction with transferrin receptor. *Cell* **93**, 111–123 (1998). 9546397 10.1016/s0092-8674(00)81151-4

[32] West, A. P. Jr. et al. Comparison of the interactions of transferrin receptor and transferrin receptor 2 with transferrin and the hereditary hemochromatosis protein HFE. *J. Biol. Chem.* **275**, 38135–38138 (2000). 11027676 10.1074/jbc.C000664200

[33] Wally, J. et al. The crystal structure of iron-free human serum transferrin provides insight into inter-lobe communication and receptor binding. *J. Biol. Chem.* **281**, 24934–24944 (2006). 16793765 10.1074/jbc.M604592200 PMC1895924

[34] Feder, J. N. et al. The hemochromatosis gene product complexes with the transferrin receptor and lowers its affinity for ligand binding. *Proc. Natl Acad. Sci. USA* **95**, 1472–1477 (1998). 9465039 10.1073/pnas.95.4.1472 PMC19050

[35] Bennett, M. J., Lebron, J. A. & Bjorkman, P. J. Crystal structure of the hereditary haemochromatosis protein HFE complexed with transferrin receptor. *Nature* **403**, 46–53 (2000). 10638746 10.1038/47417

[36] Qian, Z. M. & Tang, P. L. Mechanisms of iron uptake by mammalian cells. *Biochim. Biophys. Acta* **1269**, 205–214 (1995). 7495872 10.1016/0167-4889(95)00098-x

[37] Qian, Z. M., Tang, P. L. & Wang, Q. Iron crosses the endosomal membrane by a carrier-mediated process. *Prog. Biophys. Mol. Biol.* **67**, 1–15 (1997). 9401416 10.1016/s0079-6107(97)00009-6

[38] Nelson, N. & Harvey, W. R. Vacuolar and plasma membrane proton-adenosinetriphosphatases. *Physiol. Rev.* **79**, 361–385 (1999). 10221984 10.1152/physrev.1999.79.2.361

[39] Ohgami, R. S. et al. Identification of a ferrireductase required for efficient transferrin-dependent iron uptake in erythroid cells. *Nat. Genet* **37**, 1264–1269 (2005). 16227996 10.1038/ng1658 PMC2156108

[40] De DomenicoI, I., Ward, D. M. & Kaplan, J. Regulation of iron acquisition and storage: consequences for iron-linked disorders. *Nat. Rev. Mol. Cell Biol.* **9**, 72–81 (2008). 17987043 10.1038/nrm2295

[41] Tabuchi, M. et al. Human NRAMP2/DMT1, which mediates iron transport across endosomal membranes, is localized to late endosomes and lysosomes in HEp-2 cells. *J. Biol. Chem.* **275**, 22220–22228 (2000). 10751401 10.1074/jbc.M001478200

[42] Lane, D. J. et al. Cellular iron uptake, trafficking and metabolism: key molecules and mechanisms and their roles in disease. *Biochim. Biophys. Acta* **1853**, 1130–1144 (2015). 25661197 10.1016/j.bbamcr.2015.01.021

[43] Protchenko, O. et al. Iron chaperone poly rC Binding protein 1 protects mouse liver from lipid peroxidation and steatosis. *Hepatology* **73**, 1176–1193 (2021). 32438524 10.1002/hep.31328 PMC8364740

[44] Shaw, G. C. et al. Mitoferrin is essential for erythroid iron assimilation. *Nature* **440**, 96–100 (2006). 16511496 10.1038/nature04512

[45] Devireddy, L. R. et al. A mammalian siderophore synthesized by an enzyme with a bacterial homolog involved in enterobactin production. *Cell* **141**, 1006–1017 (2010). 20550936 10.1016/j.cell.2010.04.040 PMC2910436

[46] Richardson, D. R. et al. Mitochondrial iron trafficking and the integration of iron metabolism between the mitochondrion and cytosol. *Proc. Natl Acad. Sci. USA* **107**, 10775–10782 (2010). 20495089 10.1073/pnas.0912925107 PMC2890738

[47] Lim, J. E. et al. A mutation in *Sec15l1* causes anemia in hemoglobin deficit (*hbd*) mice. *Nat. Genet* **37**, 1270–1273 (2005). 16227995 10.1038/ng1659

[48] Chen, C. et al. Snx3 regulates recycling of the transferrin receptor and iron assimilation. *Cell Metab.* **17**, 343–352 (2013). 23416069 10.1016/j.cmet.2013.01.013 PMC3595351

[49] Muckenthaler, M. U. et al. A red carpet for iron metabolism. *Cell* **168**, 344–361 (2017). 28129536 10.1016/j.cell.2016.12.034 PMC5706455

[50] Li, L. et al. Binding and uptake of H-ferritin are mediated by human transferrin receptor-1. *Proc. Natl Acad. Sci. USA* **107**, 3505–3510 (2010). 20133674 10.1073/pnas.0913192107 PMC2840523

[51] Sakamoto, S. et al. H-ferritin is preferentially incorporated by human erythroid cells through transferrin receptor 1 in a threshold-dependent manner. *PLoS ONE* **10**, e0139915 (2015). 26441243 10.1371/journal.pone.0139915 PMC4595017

[52] Abu-Saleh, N. et al. The molecular mechanism of CD81 antibody inhibition of metastasis. *Proc. Natl Acad. Sci. USA* **120**, e2305042120 (2023). 37339209 10.1073/pnas.2305042120 PMC10293848

[53] Mazel-Sanchez, B. et al. Influenza A virus exploits transferrin receptor recycling to enter host cells. *Proc. Natl Acad. Sci. USA* **120**, e2214936120 (2023). 37192162 10.1073/pnas.2214936120 PMC10214170

[54] Radoshitzky, S. R. et al. Transferrin receptor 1 is a cellular receptor for New World haemorrhagic fever arenaviruses. *Nature* **446**, 92–96 (2007). 17287727 10.1038/nature05539 PMC3197705

[55] Gruszczyk, J. et al. Transferrin receptor 1 is a reticulocyte-specific receptor for *Plasmodium vivax*. *Science* **359**, 48–55 (2018). 29302006 10.1126/science.aan1078 PMC5788258

[56] Wang, X. et al. Transferrin receptor protein 1 is an entry factor for rabies virus. *J. Virol.* **97**, e0161222 (2023). 36779762 10.1128/jvi.01612-22 PMC9972965

[57] Cheli, V. T. et al. Transferrin receptor is necessary for proper oligodendrocyte iron homeostasis and development. *J. Neurosci.* **43**, 3614–3629 (2023). 36977582 10.1523/JNEUROSCI.1383-22.2023 PMC10198458

[58] Parrow, N. L. & Fleming, R. E. Transferrin receptor 1: keeper of HFE. *Blood* **141**, 332–333 (2023). 36701171 10.1182/blood.2022018740 PMC9936298

[59] Das, B. K. et al. Transferrin receptor 1-mediated iron uptake regulates bone mass in mice via osteoclast mitochondria and cytoskeleton. *eLife* **11**, e73539 (2022). 35758636 10.7554/eLife.73539 PMC9352353

[60] Sehlin, D. & Syvänen, S. MINC faculty. Engineered antibodies: new possibilities for brain PET? *Eur. J. Nucl. Med Mol. Imaging* **46**, 2848–2858 (2019). 31342134 10.1007/s00259-019-04426-0 PMC6879437

[61] Yuan, Z. et al. Rational design of engineered H-ferritin nanoparticles with improved siRNA delivery efficacy across an in vitro model of the mouse BBB. *Nanoscale* **14**, 6449–6464 (2022). 35416195 10.1039/d1nr07880a

[62] Stocki, P. et al. Blood–brain barrier transport using a high affinity, brain-selective VNAR antibody targeting transferrin receptor 1. *FASEB J.* **35**, e21172 (2021). 33241587 10.1096/fj.202001787R

[63] Leoh, L. S. et al. Insights into the effector functions of human IgG3 in the context of an antibody targeting transferrin receptor 1. *Mol. Immunol.* **67**, 407–415 (2015). 26232328 10.1016/j.molimm.2015.07.001 PMC4636009

[64] Kawabata, H. et al. Molecular cloning of transferrin receptor 2. A new member of the transferrin receptor-like family. *J. Biol. Chem.* **274**, 20826–20832 (1999). 10409623 10.1074/jbc.274.30.20826

[65] Roetto, A., Mezzanotte, M. & Pellegrino, R. M. The functional versatility of transferrin receptor 2 and its therapeutic value. *Pharm. (Basel)* **11**, 115 (2018). 10.3390/ph11040115 PMC6316356 30360575

[66] Mastroberardino, P. G. et al. A novel transferrin/TfR2-mediated mitochondrial iron transport system is disrupted in Parkinson’s disease. *Neurobiol. Dis.* **34**, 417–431 (2009). 19250966 10.1016/j.nbd.2009.02.009 PMC2784936

[67] Fleming, R. E. et al. Transferrin receptor 2: continued expression in mouse liver in the face of iron overload and in hereditary hemochromatosis. *Proc. Natl Acad. Sci. USA* **97**, 2214–2219 (2000). 10681454 10.1073/pnas.040548097 PMC15780

[68] Robb, A. & Wessling-Resnick, M. Regulation of transferrin receptor 2 protein levels by transferrin. *Blood* **104**, 4294–4299 (2004). 15319276 10.1182/blood-2004-06-2481

[69] Chang, Y. Z., Duan, S. L. & Qian, Z. M. Transferrin receptor 2: function and relevant disorders. *Prog. Biophys. Biochem* **30**, 533–536 (2003).

[70] Kawabata, H. et al. Expression of transferrin receptor 2 in normal and neoplastic hematopoieticcells. *Blood* **98**, 2714–2719 (2001). 11675342 10.1182/blood.v98.9.2714

[71] Chen, J. & Enns, A. CD81 promotes both the degradation of transferrin receptor 2 (TfR2) and the Tfr2-mediated maintenance of hepcidin expression. *J. Biol. Chem.* **290**, 7841–7850 (2015). 25635054 10.1074/jbc.M114.632778 PMC4367283

[72] Kawabata, H. et al. Transferrin receptor 2-alpha supports cell growth both in iron-chelated cultured cells and in vivo. *J. Biol. Chem.* **275**, 16618–16625 (2000). 10748106 10.1074/jbc.M908846199

[73] Fleming, R. E. et al. Targeted mutagenesis of the murine transferrin receptor-2 gene produces hemochromatosis. *Proc. Natl Acad. Sci. USA* **99**, 10653–10658 (2002). 12134060 10.1073/pnas.162360699 PMC125003

[74] Chen, J. & Enns, C. A. Hereditary hemochromatosis and transferrin receptor 2. *Biochim. Biophys. Acta* **1820**, 256–263 (2012). 21864651 10.1016/j.bbagen.2011.07.015 PMC3234335

[75] Kawabata, H. Transferrin and transferrin receptors update. *Free Radic. Biol. Med* **133**, 46–54 (2019). 29969719 10.1016/j.freeradbiomed.2018.06.037

[76] Giannetti, A. M. & Björkman, P. J. HFE and transferrin directly compete for transferrin receptor in solution and at the cell surface. *J. Biol. Chem.* **279**, 25866–25875 (2004). 15056661 10.1074/jbc.M401467200

[77] Babitt, J. L. et al. Bone morphogenetic protein signaling by hemojuvelin regulates hepcidin expression. *Nat. Genet* **38**, 531–539 (2006). 16604073 10.1038/ng1777

[78] Wang, C. Y. & Babitt, J. L. Liver iron sensing and body iron homeostasis. *Blood* **133**, 18–29 (2019). 30401708 10.1182/blood-2018-06-815894 PMC6318427

[79] Wallace, D. F. et al. Combined deletion of Hfe and transferrin receptor 2 in mice leads to marked dysregulation of hepcidin and iron overload. *Hepatology* **50**, 1992–2000 (2009). 19824072 10.1002/hep.23198

[80] Corradini, E. et al. Serum and liver iron differently regulate the bone morphogenetic protein 6 (BMP6)-SMAD signaling pathway in mice. *Hepatology* **54**, 273–284 (2011). 21488083 10.1002/hep.24359 PMC3277401

[81] Olivari, V. et al. A single approach to targeting transferrin receptor 2 corrects iron and erythropoietic defects in murine models of anemia of inflammation and chronic kidney disease. *Kidney Int.* **104**, 61–73 (2023). 36990212 10.1016/j.kint.2023.03.012

[82] Nai, A. et al. The second transferrin receptor regulates red blood cell production in mice. *Blood* **125**, 1170–1179 (2015). 25499454 10.1182/blood-2014-08-596254 PMC4399753

[83] Rauner, M. et al. Transferrin receptor 2 controls bone mass and pathological bone formation via BMP and Wnt signaling. *Nat. Metab.* **1**, 111–124 (2019). 30886999 10.1038/s42255-018-0005-8 PMC6420074

[84] Ledesma-Colunga, M. G. et al. Transferrin receptor 2 deficiency promotes macrophage polarization and inflammatory arthritis. *Redox Biol.* **60**, 102616 (2023). 36746004 10.1016/j.redox.2023.102616 PMC9932570

[85] Modun, B., Kendall, D. & Williams, P. Staphylococci express a receptor for human transferrin: identification of a 42-kilodalton cell wall transferrin-binding protein. *Infect. Immun.* **62**, 3850–3858 (1994). 8063401 10.1128/iai.62.9.3850-3858.1994 PMC303040

[86] Modun, B., Morrissey, J. & Williams, P. The staphylococcal transferrin receptor: a glycolytic enzyme with novel functions. *Trends Microbiol* **8**, 231–237 (2000). 10785640 10.1016/s0966-842x(00)01728-5

[87] Raje, C. I. et al. The macrophage cell surface glyceraldehyde-3-phosphate dehydrogenase is a novel transferrin receptor. *J. Biol. Chem.* **282**, 3252–3261 (2007). 17121833 10.1074/jbc.M608328200

[88] Boradia, V. M., Raje, M. & Raje, C. I. Protein moonlighting in iron metabolism: glyceraldehyde-3-phosphate dehydrogenase (GAPDH). *Biochem. Soc. Trans.* **42**, 1796–1801 (2014). 25399609 10.1042/BST20140220

[89] Boradia, V. M. et al. *Mycobacterium tuberculosis* acquires iron by cell-surface sequestration and internalization of human holo-transferrin. *Nat. Commun.* **5**, 4730 (2014). 25163484 10.1038/ncomms5730

[90] Kumar, S. et al. Characterization of glyceraldehyde-3-phosphate dehydrogenase as a novel transferrin receptor. *Int J. Biochem. Cell Biol.* **44**, 189–199 (2012). 22062951 10.1016/j.biocel.2011.10.016

[91] Lazarev, V. F., Guzhova, I. V. & Margulis, B. A. Glyceraldehyde-3-phosphate dehydrogenase is a multifaceted therapeutic target. *Pharmaceutics* **12**, 416 (2020). 32370188 10.3390/pharmaceutics12050416 PMC7285110

[92] Nombela, C., Gil, C. & Chaffin, W. L. Non-conventional protein secretion in yeast. *Trends Microbiol.* **14**, 15–21 (2006). 16356720 10.1016/j.tim.2005.11.009

[93] Fugier, E. et al. The glyceraldehyde-3-phosphate dehydrogenase and the small GTPase Rab 2 are crucial for *Brucella* replication. *PLoS Pathog.* **5**, e1000487 (2009). 19557163 10.1371/journal.ppat.1000487 PMC2695806

[94] Malhotra, H. et al. Moonlighting protein glyceraldehyde-3-phosphate dehydrogenase: a cellular rapid-response molecule for maintenance of iron homeostasis in hypoxia. *Cell Physiol. Biochem.* **52**, 517–531 (2019). 30897319 10.33594/000000037

[95] Jeffery, C. J. Moonlighting proteins. *Trends Biochem. Sci.* **24**, 8–11 (1999). 10087914 10.1016/s0968-0004(98)01335-8

[96] Sirover, M. A. On the functional diversity of glyceraldehyde-3-phosphate dehydrogenase: biochemical mechanisms and regulatory control. *Biochim. Biophys. Acta* **1810**, 741–751 (2011). 21640161 10.1016/j.bbagen.2011.05.010

[97] Sen, N. et al. Nitric oxide-induced nuclear GAPDH activates p300/CBP and mediates apoptosis. *Nat. Cell Biol.* **10**, 866–873 (2008). 18552833 10.1038/ncb1747 PMC2689382

[98] Tupta, B. et al. GAPDH is involved in the heme-maturation of myoglobin and hemoglobin. *FASEB J.* **36**, e22099 (2022). 34972240 10.1096/fj.202101237RR PMC9239731

[99] Dautry-Varsat, A., Ciechanover, A. & Lodish, H. F. pH and the recycling of transferrin during receptor-mediated endocytosis. *Proc. Natl Acad. Sci. USA* **80**, 2258–2262 (1983). 6300903 10.1073/pnas.80.8.2258 PMC393798

[100] Griffiths, J. & Shaw, S. Glyceraldehyde-phosphate dehydrogenase (total and isoenzyme activity) in the early diagnosis of myocardial infarction. *Clin. Chem.* **23**, 245–249 (1977). 832386

[101] Sheokand, N. et al. Secreted glyceraldehye-3-phosphate dehydrogenase is a multifunctional autocrine transferrin receptor for cellular iron acquisition. *Biochim. Biophys. Acta* **1830**, 3816–3827 (2013). 23541988 10.1016/j.bbagen.2013.03.019

[102] Kozyraki, R. et al. Megalin-dependent cubilin-mediated endocytosis is a major pathway for the apical uptake of transferrin in polarized epithelia. *Proc. Natl Acad. Sci. USA* **98**, 12491–12496 (2001). 11606717 10.1073/pnas.211291398 PMC60081

[103] Kozyraki, R. et al. The human intrinsic factor-vitamin B_12_ receptor, cubilin: molecular characterization and chromosomal mapping of the gene to 10p within the autosomal recessive megaloblastic anemia (MGA1) region. *Blood* **91**, 3593–3600 (1998). 9572993

[104] Moestrup, S. K. et al. The intrin sic factor-vitamin B_12_ receptor and target of teratogenic antibodies is a megalin-binding peripheral membrane protein with homology to developmental proteins. *J. Biol. Chem.* **273**, 5235–5242 (1998). 9478979 10.1074/jbc.273.9.5235

[105] Kozyraki, R. et al. The intrinsic factor-vitamin B_12_ receptor, cubilin, is a high-affinity apolipoprotein A-I receptor facilitating endocytosis of high-density lipoprotein. *Nat. Med.* **5**, 656–661 (1999). 10371504 10.1038/9504

[106] Christensen, E. I., Nielsen, R. & Birn, H. From bowel to kidneys: the role of cubilin in physiology and disease. *Nephrol. Dial. Transpl.* **28**, 274–281 (2013). 10.1093/ndt/gfs565 23291372

[107] Fyfe, J. C. et al. The functional cobalamin (vitamin B_12_)-intrinsic factor receptor is a novel complex of cubilin and amnionless. *Blood* **103**, 1573–1579 (2004). 14576052 10.1182/blood-2003-08-2852

[108] Andersen, C. B. F. et al. Structural basis for receptor recognition of vitamin-B_12_-intrinsic factor complexes. *Nature* **464**, 445–448 (2010). 20237569 10.1038/nature08874

[109] Christensen, E. I. & Birn, H. Megalin and cubilin: multifunctional endocytic receptors. *Nat. Rev. Mol. Cell Biol.* **3**, 256–266 (2002). 11994745 10.1038/nrm778

[110] Christensen, E. I. et al. Endocytic receptors in the renal proximal tubule. *Physiol. (Bethesda)* **27**, 223–236 (2012). 10.1152/physiol.00022.2012 22875453

[111] Prabakaran, T. et al. Cubilin is expressed in rat and human glomerular podocytes. *Nephrol. Dial. Transpl.* **27**, 3156–3159 (2012). 10.1093/ndt/gfr794 22337902

[112] Saito, A. et al. Complete cloning and sequencing of rat gp330/“megalin,” a distinctive member of the low density lipoprotein receptor gene family. *Proc. Natl Acad. Sci. USA* **91**, 9725–9729 (1994). 7937880 10.1073/pnas.91.21.9725 PMC44889

[113] Christensen, E. I. & Birn, H. Megalin and cubilin: synergistic endocytic receptors in renal proximal tubule. *Am. J. Physiol. Ren. Physiol.* **280**, F562–F573 (2001). 10.1152/ajprenal.2001.280.4.F562 11249847

[114] Kerjaschki, D. & Farquhar, M. G. The pathogenic antigen of Heymann nephritis is a membrane glycoprotein of the renal proximal tubule brush border. *Proc. Natl Acad. Sci. USA* **79**, 5557–5561 (1982). 6752952 10.1073/pnas.79.18.5557 PMC346943

[115] Kerjaschki, D. & Farquhar, M. G. Immunocytochemical localization of the Heymann nephritis antigen (GP330) in glomerular epithelial cells of normal Lewis rats. *J. Exp. Med.* **157**, 667–686 (1983). 6337231 10.1084/jem.157.2.667 PMC2186920

[116] Raychowdhury, R. et al. Autoimmune target in Heymann nephritis is a glycoprotein with homology to the LDL receptor. *Science* **244**, 1163–1165 (1989). 2786251 10.1126/science.2786251

[117] Weiss, A. et al. Orchestrated regulation of iron trafficking proteins in the kidney during iron overload facilitates systemic iron retention. *PLoS ONE* **13**, e0204471 (2018). 30321179 10.1371/journal.pone.0204471 PMC6188744

[118] Smith, C. P. et al. Fenton, proximal tubule transferrin uptake is modulated by cellular iron and mediated by apical membrane megalin–cubilin complex and transferrin receptor 1. *J. Biol. Chem.* **294**, 7025–7036 (2019). 30833328 10.1074/jbc.RA118.006390 PMC6497946

[119] Odera, K., Goto, S. & Takahashi, R. Age-related change of endocytic receptors megalin and cubilin in the kidney in rats. *Biogerontology* **8**, 505–515 (2007). 17453355 10.1007/s10522-007-9093-7

[120] Figueira, F. M. et al. Diabetic rats present higher urinary loss of proteins and lower renal expression of megalin, cubilin, ClC-5, and CFTR. *Physiol. Rep.* **5**, e13335 (2017). 28676554 10.14814/phy2.13335 PMC5506523

[121] Christensen, E. I. et al. Loss of chloride channel ClC-5 impairs endocytosis by defective trafficking of megalin and cubilin in kidney proximal tubules. *Proc. Natl Acad. Sci. USA* **100**, 8472–8477 (2003). 12815097 10.1073/pnas.1432873100 PMC166253

[122] Bednarz, A. et al. Exacerbation of neonatal hemolysis and impaired renal iron handling in heme oxygenase 1-deficient mice. *Int J. Mol. Sci.* **21**, 7754 (2020). 33092142 10.3390/ijms21207754 PMC7589678

[123] Kalantry, S. et al. The amnionless gene, essential for mouse gastrulation, encodes a visceral-endoderm- specific protein with an extracellular cysteine-rich domain. *Nat. Genet.* **27**, 412–416 (2001). 11279523 10.1038/86912

[124] Tanner, S. M. et al. Amnionless, essential for mouse gastrulation, is mutated in recessive hereditary megaloblastic anemia. *Nat. Genet.* **33**, 426–429 (2003). 12590260 10.1038/ng1098

[125] Fyfe, J. C. et al. Defective brush-border expression of intrinsic factor-cobalamin receptor in canine inherited intestinal cobalamin malabsorption. *J. Biol. Chem.* **266**, 4489–4494 (1991). 1999430

[126] Wolff, N. A. et al. Megalin-dependent internalization of cadmium-metallothionein and cytotoxicity in cultured renal proximal tubule cells. *J. Pharm. Exp. Ther.* **318**, 782–791 (2006). 10.1124/jpet.106.102574 16690719

[127] Barone, R. et al. Endocytosis of the somatostatin analogue, octreotide, by the proximal tubule-derived opossum kidney (OK) cell line. *Kidney Int.* **67**, 969–976 (2005). 10.1111/j.1523-1755.2005.00160.x 15698435

